# Impact of effectiveness information format on patient choice of therapy and satisfaction with decisions about chronic disease medication: the "Influence of intervention Methodologies on Patient Choice of Therapy (IMPACT)" cluster-randomised trial in general practice

**DOI:** 10.1186/1472-6963-13-76

**Published:** 2013-02-25

**Authors:** Charlotte Gry Harmsen, Dorte Ejg Jarbøl, Jørgen Nexøe, Henrik Støvring, Dorte Gyrd-Hansen, Jesper Bo Nielsen, Adrian Edwards, Ivar Sønbø Kristiansen

**Affiliations:** 1 Research Unit of General Practice, University of Southern Denmark, Southern Denmark, Denmark; 2 Department of Public Health, Biostatistics, Aarhus University, Aarhus, Denmark; 3 Institute of Public Health, University of Southern Denmark, Southern Denmark, Denmark; 4 Cochrane Institute of Primary Care & Public Health, School of Medicine, Cardiff University, Wales, UK; 5 Department of Health Management and Health Economics, University of Oslo, Oslo, Norway

**Keywords:** RCT, Shared decision making, Risk communication, Prognosis, Absolute risk reduction, Prolongation of life, Cardiovascular disease, Primary prevention, Health behaviour, General practice

## Abstract

**Background:**

Risk communication is an integral part of shared decision-making in health care. In the context of interventions for chronic diseases it represents a particular challenge for all health practitioners. By using two different quantitative formats to communicate risk level and effectiveness of a cholesterol-lowering drug, we posed the research question: how does the format of risk information influence patients’ decisions concerning therapy, patients’ satisfaction with the communication as well as confidence in the decision. We hypothesise that patients are less prone to accept therapy when the benefits of long-term intervention are presented in terms of prolongation of life (POL) in months compared to the absolute risk reduction (ARR). We hypothesise that patients presented with POL will be more satisfied with the communication and confident in their decision, suggesting understanding of the time-related term.

**Methods/Design:**

In 2009 a sample of 328 general practitioners (GPs) in the Region of Southern Denmark was invited to participate in a primary care-based clinical trial among patients making real-life clinical decisions together with their GP. Interested GPs were cluster-randomised to inform patients about cardiovascular disease (CVD) risk and the effectiveness of statin therapy using either POL or ARR. The GPs attended a training session before informing their patients. Before training and after the trial period they received a questionnaire about their attitudes to risk communication and the use of numerical information. Patients’ redemptions of statin prescriptions will be registered in a regional prescription database to evaluate a possible association between redemption rates and effectiveness format. The Combined Outcome Measure for Risk Communication And Treatment Decision Making Effectiveness (COMRADE) questionnaire will be used to measure patients’ confidence and satisfaction with the risk communication immediately after the conversation with their GPs.

**Discussion:**

This randomised clinical trial compares the impact of two effectiveness formats on real-life risk communication between patients and GPs, including affective patient outcomes and actual choices about acceptance of therapy. Though we found difficulties in recruiting GPs, according to the study protocol we have succeeded in engaging sufficient GPs for the trial, enabling us to perform the planned analyses.

**Trial registration:**

ClinicalTrials.gov Protocol Registration System http://ww.clinicaltrials.gov/NCT01414751

## Background

In a time when shared decision-making is widely recommended for clinical practice, risk communication – an integral component of shared decision-making – has become an important factor in the doctor-patient encounter [1-4]. This is especially true for patients confronted with the risk of a possible future chronic disease such as cardiovascular disease. Here the decision concerns lifestyle changes or initiation of long-term preventive medication to reduce the risk of adverse outcomes. Pharmaceutical interventions offer well-documented benefits at the population level. To the individual patient, however, the immediate clinical effect may be side-effects, whereas benefits may not be possible to identify or measure directly. For shared decision-making to be achieved risk communication is crucial. Effective risk communication can enhance knowledge, involvement in decisions about interventions, autonomy and empowerment of patients [5]. In contrast, poor communication can lead to anxiety, lack of confidence in the doctor, and to decisions based on misinterpreted information and inadequate communication.

Communicating risk requires skills not always mastered by patients or doctors alike [6-11]. Skill acquisition is of particular importance when the different numerical presentations of risk are communicated between doctors and their patients. The standard quantitative effectiveness formats are

•RRR - relative risk reduction

•ARR - absolute risk reduction

•NNT - number needed to treat

•POL – prolongation of life (or disease-/symptom-free survival) (Table 1).

**Table 1:** Four different ways of expressing the effect of preventive cholesterol-lowering therapy to a hypercholesterolaemic person


**Format**	**Examples of ways of expression**
RRR	If a person with high cholesterol level like you takes the medicine, the risk of dying from a heart attack will be reduced by 33% within the next 10 years
ARR	If people with high cholesterol level like you do not take the medicine on average 15 out of 100 people, 15%, will die of a heart attack within the next 10 years. However, if people take the medicine on average 10 out of 100, 10%, will die of a heart attack over the next 10 years. That means that on average 5% fewer persons will die
NNT	If 20 people with high cholesterol level like you take the medicine, there will on average be one more alive after 10 years than if they do not take the medicine
POL	If people with high cholesterol level like you take the medicine, for the rest of their lives they will on average live 8 months longer than if they do not take medicine

Several studies have evaluated these formats in risk communication and how they can be used in the doctor-patient encounter [12-16]. The choice of format depends on various factors, e.g. whether the format is perceived to be easy to understand; whether it is correctly understood; whether the format meets patients’ information needs; whether patients receive enough information to make optimal decisions [17,18]; and how understanding of the format may be affected by factors like age, gender, or educational level. By presenting risk in different ways, framing effects may influence how it is perceived [19]. A body of research demonstrates that even when conveying essentially the same information, patients, doctors and policy-makers are influenced by differences in format, including the specific wording and presentation (e.g. percentages versus natural frequencies) [15,20]. Pictorial representations seem to be better understood than natural frequencies, and natural frequencies better than percentages [15]. A Danish study [13] has indicated that respondents in a survey tend to be more consistent in their decisions when information is presented in terms of ARR rather than RRR or NNT. Absolute risk is often used by GPs when they communicate risk with their patients and discuss cardiovascular prevention. The GPs often make use of the Systematic Coronary Risk Assessment chart (SCORE) [21] or national modified algorithms [22-26]. The SCORE algorithm calculates a person’s 10-year statistical risk of fatal cardiovascular disease by means of the absolute risk in percentages, based on the patient’s gender, age, smoking status, systolic blood pressure and total-cholesterol.

The use of POL in effectiveness communication is a relatively new format of risk reduction. A study of osteoporosis interventions [27] found that lay people seem to understand POL in the sense that they were able to discriminate between different levels of effectiveness when presented in terms of disease-free survival (i.e. “postponement” of hip fracture), but not in terms of NNT. Similar results have been shown in later studies [14,28,29]. The studies indicate that the formats representing the most comprehensible effectiveness may be ARR and POL. A recent Danish study [30] proposes a modified algorithm which is based on the same stratifications as the SCORE model. It presents the estimated ten-year mortality risk without statin treatment and the estimated ARR in percentages with treatment, and with inclusion of non-CVD mortality to provide estimates of all-cause mortality. Further, the modified algorithm provides estimates of the remaining life expectancy without treatment and the POL in months if treated with a statin. Providing risk and extension estimates with and without treatment makes it possible to inform patients about the expected gain on offer. The modified algorithm (see Additional file 1) forms the basis of the intervention in the IMPACT trial described in this study protocol.

Many studies on risk communication explore the conceptual development as to comprehension and presentation of risk [31] or how lay people, doctors or policy makers choose treatment in hypothetical situations. Studies have involved patients [6], but rarely for decisions about their own therapy. It is therefore unclear how closely the findings in hypothetical situations accord with real-life decisions. To the best of our knowledge, no randomised practice-based studies have yet explored how real patients’ real-life decisions are influenced by different risk formats.

## Objective and hypotheses

The primary objective of the IMPACT trial was in a randomised design to explore how risk communication using ARR versus POL as effectiveness format may affect a range of endpoints: patients’ decision about redemption of statin prescriptions, patients’ satisfaction with the communication in the consultation and patients’ confidence in their decision. Secondarily, we aimed to study the impact of teaching the GPs to use the respective effectiveness formats on GPs’ opinions and experiences in relation to statin prescribing and risk communication. We chose to use cholesterol-lowering therapy with statins, as hypercholesterolaemia is a well-defined condition, and because statins as the predominant treatment of hypercholesterolaemia are used for this condition only, ensuring that prescribed treatment is used for CVD prevention.

Based on previous research [13,14,27,32] we hypothesise that patients understand POL better than ARR because most people have more experience with assessment of differences in time than reduced percentages in risk.

## Methods

### Design and setting

This cluster-randomised practice-based study has been implemented among GPs (single-handed or partnership practices) in the Region of Southern Denmark and their candidate patients for primary CVD prevention with statins.

### Randomisation

Randomisation took place at the level of general practices and allocated the practices to one of two different ways of presenting the effectiveness of cholesterol-lowering therapy. At an afternoon meeting with several practices gathered, randomisation was based on grouping of doctors in pairs using geographical location as first criterion, then type of practice (single-handed or partnership). In each pair one GP was allocated to ARR, and the other to POL based on a random-number computer-generated table prepared before recruitment of general practices.

When visiting individual single-handed practices during working hours a concealed random-number computer-generated table with block randomisation of the two formats in groups of 4 was used for allocation, and an alternative list used for partnership practices.

### Participants

#### GPs

In August 2009 the Regional Council of Southern Denmark, provided us with a list of 328 GPs in 13 municipalities in the Region of Southern Denmark (1.2 million inhabitants). At first we had planned to invite only GPs from two of the municipalities, Kolding and Fredericia (a total of 93 GPs), to obtain the number of practices needed. However, we did not succeed in recruiting enough GPs, and in October 2009 we consequently decided to invite all GPs on the list. We invited GPs to participate in a clinical trial concerning use of quantitative effectiveness formats in risk communication in health prevention consultations (typical consultations at which a GP would review risk factors, levels of risk and potential benefits of lifestyle or pharmacological interventions to manage risk). Subsequently, the project manager (CGH) contacted the GPs by phone and provided further details about the trial. During this phone call, the GPs were briefly informed about practicalities if they accepted to participate.

**Figure 1:**
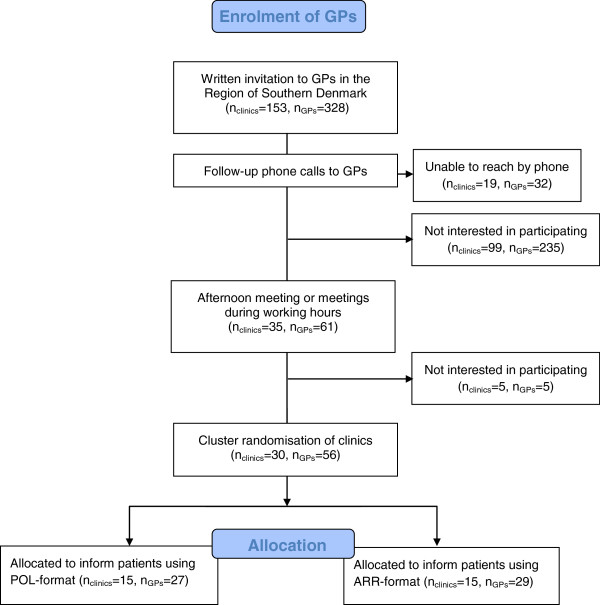
Flowchart of GP enrolment.

A total of 30 practices (18 single-handed and 12 partnership practices) were included in the study (Figure 1).

#### Patients

The GPs have been asked to invite patients to participate in the trial if the patients have had blood samples taken for measurement of total-cholesterol and subsequently returning for a health prevention consultation. Patients aged 40 to 69 years with a total-cholesterol level above 4 mmol/L (155 mg/dL) will be eligible. This level is in accordance with Danish national guidelines for prevention of ischaemic CVD [25]. Patients will be excluded if they have diabetes, known CVD (including stroke, angina or myocardial infarction), if they have already received cholesterol-lowering medication, or if they are not fluent in Danish. The patients will be informed verbally that the trial concerns the way GPs communicate risk with their patients and the patient’s perception of the risk communication.

The patients will receive a written description of the project including a letter of consent to be signed by the patient before entering the trial. The GPs will fill out a specially designed form with information on the individual patient’s age, gender, systolic blood pressure, total-cholesterol, and whether the patient consents to participate.

### Intervention

The GPs accepting to participate were invited to either a joint afternoon information meeting or a meeting during working hours in the GPs’ clinics. The purpose of the meetings was to instruct the GPs in the use of either ARR or POL. The GPs who attended the afternoon meeting participated in a two-hour session. The first part of the session featured a Power Point presentation by the project manager (CGH) showing the design of the project. This was followed by randomisation of the attending GPs (n=7) to either ARR or POL. The two groups then had separate information sessions taught by two senior researchers (both GPs). The GPs were then, based on the randomisation, instructed to stick to the specifically allocated effectiveness format when evaluating the measured total-cholesterol with their patients using specially designed algorithms for risk assessment (see Additional file 1). The GPs were told that the focus in the conversation with their patients should be on total-cholesterol and primary prevention of hypercholesterolaemia. All GPs who agreed to participate in the trial were asked to complete a questionnaire before they began to recruit patients. The questionnaire consisted of seven items concerning the individual GP’s preferences and experiences with the use of quantitative formats when discussing preventive therapies with their patients, and six items concerning characteristics of the GP’s practice and workload.

The GPs received a light meal provided by the project as well as reimbursement for lost income for the time spent attending the meeting. Additional afternoon meetings were offered, but not implemented, because the GPs were either not interested or unable to participate. GPs who were interested, but unable to participate in an afternoon meeting, were offered a meeting during working hours in their clinics. These sessions were conducted by the project manager.

Patients interested in participating in the trial will receive a questionnaire containing 35 items. The questionnaire includes a translated version of the British COMRADE instrument [33-39] consisting of two subscales. The subscale ‘confidence in decision’ comprises 10 items and focuses on the exchange and content of information as seen from the patient’s perspective. The subscale ‘risk communication’ comprises 10 items concerning the emotional evaluation of the communication. According to proposed guidelines [40-42] the instrument has been forward-translated from English into Danish and back-translated into English, and finally tested for content validity. The remaining items of the questionnaire concern sociodemographic issues (education, household income, marital status), experience with cardiovascular disease in the patient’s family and self-rated health. Ideally, the questionnaire is to be answered immediately after the consultation for the patients to have a clear memory of the consultation. The patients are to return the questionnaire in a prepaid envelope addressed to the research group. Patients who do not return the questionnaire will receive up to two postal reminders.

After patient recruitment has ended, we will ask the GPs to complete a second questionnaire concerning their experiences with using the agreed quantitative effectiveness format they have been allocated when discussing preventive therapies with their patients.

### Outcomes

The primary outcome measure is patients’ redemptions of statin prescriptions based on data from Odense University Pharmaco-Epidemiological Database (OPED) during a three-month period after inclusion (the day the patient receives risk information and consents to participate). The three months will allow for time to consider and adopt lifestyle changes before initiating cholesterol-lowering therapy in accordance with Danish guidelines for primary prevention of CVD [22].

OPED covers all individuals in the Region of Southern Denmark and captures information on redemption of reimbursed drugs. Based on the Anatomical Therapeutic Chemical (ATC) classification [43], OPED has information on the number of packages and number of Defined Daily Doses (DDDs) dispensed, prescriber identification, and date of prescription.

The secondary endpoints are patients’ understanding of the provided risk information, their perception of involvement, confidence in the decision made, and satisfaction with the communication. Here, we will use the COMRADE instrument [33-39].

### Sample size

We based the sample size calculation on expected redemptions of statin prescriptions in the two groups. We expected 80% redemption in the ARR group and 60% in the POL group. This would require 2 groups of each 91 patients to obtain an 80% power with 5% significance in a non-clustered study. Due to clustering, and assuming an intra-cluster correlation of 0.1, we estimated a need for a total of 346 patients for 80% power and 5% significance. We included some buffer due to possible drop-outs and ended up with a total of 380 patients expected to be included by a total of 40 practices.

### Statistical methods

We will use descriptive statistics and analyse differences between groups by means of multilevel logistic and linear regression modeling, to account for possible clustering effects. The models will have three nested levels: patient-, GP-, and practice-level. Regression models will be used to test for differences between the POL- and the ARR-arm in patients’ redemption rates and the COMRADE outcomes, patients’ confidence and satisfaction with communication, respectively. Three of the COMRADE items focus on patients’ ease of understanding information, patients’ satisfaction with the information given, and adequacy of information about issues important for the decision, respectively. We will conduct separate analyses on each of these three items. In addition to the explanatory variable information format (POL *versus* ARR) we will include variables which may either be independently associated with the outcome variable or which have been chosen *a priori* from considerations based on expectations and knowledge within the field. These variables include patients’ baseline risk, patients’ history of angina, impaired circulation or hypertension, patients’ marital status, age and gender, GPs’ professional experience (number of years in general practice), GPs’ prior knowledge of the allocated format, GPs’ workload, and an affiliation between patient and GP of more than five years. Missing responses will be excluded from the analyses.

All analyses will be performed in STATA version 11 (STATACorp, College Station, TX, USA) [44].

### Ethical aspects

The trial has been conducted in accordance with Danish legislation and was approved by the Research Ethics Committee of the Region of Southern Denmark (project identification number S-20090034) and reported to the Danish Data Protection Agency (file number 2009-41-3208). The trial has been registered at ClinicalTrials.gov ( http://ww.clinicaltrials.gov/NCT01414751).

## Discussion

### Summary of objective and design

The objective of the IMPACT trial was in a cluster-randomised design to compare two different effectiveness formats, ARR and POL, used in real-life clinical settings in the risk communication between GPs and patients. Though there were challenges in the recruitment of GPs, we did estimate the recruited GPs to be sufficient to include patients for the trial, and allowing the planned analyses.

#### Strengths and limitations of the study design

Unlike the majority of trials within the field of risk communication, this trial is based on real-life patients making real decisions about interventions to reduce the risk of a disease, and reflects on real-life consultations. This implies that we will not know exactly how communication between the GPs and their patients takes place. We will not know whether every patient will have the respective effectiveness format explained; nor can we rule out selection bias in the GPs’ inclusion of patients. However, we have chosen a cluster-randomised design, where the intervention has been randomised at the practice level rather than at the level of individual patients. By allocating at this level and enabling the individual GPs to use only one of the effectiveness formats, there will be limited or no risk of the contaminating effect of the GPs mixing up the two formats and communicating the wrong information to their patients. Further, because the modified algorithm for ARR and POL, respectively [30], has not been freely available at the time of the study, there has been little risk of “information contamination” between the GPs.

A limitation may be that only little information will be collected about the content of the patient-GP encounters, except for essential characteristics of the patient and GP concerned. Qualitative interviews may have indicated issues that we would not be aware of.

We estimated a need for 40 practices to recruit sufficient patients. However, we had unexpected challenges recruiting the GPs. Of the 328 GPs, who received written invitations and follow-up phone-calls, only 30 practices ended up engaging in the trial. This sparse engagement calls for reflections as to the risk of performing the study within a selected group of GPs and limited generalisability.

In the protocol development we had expected that GPs might be difficult to recruit. First, the workload of GPs has increased considerably in recent years due to the transfer of some treatment regimens and follow-ups from the secondary to the primary level (especially concerning chronic diseases), and intensified efforts concerning health promotion. Further, GPs run their practices as private business, and time used for engagement in research means less time for consultations and thus reduced income. Providing the GPs with fees corresponding to the time spent both on meetings and patient communication in connection with the study we aimed at compensating income losses some, but still recruitment was difficult.

Nevertheless, a considerable number of the GPs, whether recruited or not, expressed a need for research in the area of risk communication and development of tools for use in health prevention consultations.

#### Implications

With the results of the IMPACT trial we aim to evaluate the impact of two effectiveness formats on patients’ decision-making concerning primary preventive therapy. The results may contribute in suggesting how risk communication in the GP-patient encounter and patients’ satisfaction with the decision-making could be optimised, if using effectiveness formats when discussing risk and possible benefits of preventive interventions.

This trial, with a focus on the impact of effectiveness formats on real patients, will prepare the ground for future research within the field of risk communication between health professionals and their patients, and on how real patients make real decisions. Factors other than effectiveness information may account for patients’ risk perception and influence patients’ decision-making. An important issue remaining to be explored within the area of shared decision-making and primary preventive therapy is real patients’ perception of risk, both in general and when concerning their own risk, specifically when informed about prognosis of disease. It would be particularly important to know how patients’ as well as their GPs’ perception of risk may influence decisions concerning choice of therapy and adherence in the sense of the robustness of patients’ decision-making with regard to whether or not to accept therapy. Knowledge about how patients and their GPs reflect upon and understand risk information in relation to making decisions that they would adhere to could increase cost-effectiveness of treatment, if appropriate interventions were designed based on such evidence.

## Abbreviations

ARR: Absolute risk reduction;ATC: Anatomical Therapeutic Chemical;COMRADE: Combined Outcome Measure for Risk Communication And Treatment Decision Making Effectiveness;CVD: Cardiovascular disease;DDD: Defined daily dose;GP: General practitioner;IMPACT: Influence of intervention methodologies on patient choice of therapy;NNT: Number needed to treat;OPED: Odense university pharmaco-epidemiological database;POL: Prolongation of life;RRR: Relative risk reduction;SCORE: Systematic coronary risk assessment chart

## Competing interests

The authors declared that they have no competing interest.

## Authors’ contributions

CGH contributed to study design and development of intervention. She drafted the study protocol and coordinates the implementation of the intervention. HS contributes to the statistics, and together with DEJ, JN, HS, DGH, JBN, and ISK conceived the idea of the study and developed the design. AE contributed to the study design, the evaluation and implementation of COMRADE in a Danish setting. DEJ leads the clinical trial and contributes to the coordination of the implementation. All authors contributed to the drafting of the study protocol. All authors read and approved the final manuscript.

## Pre-publication history

The pre-publication history for this paper can be accessed here:

http://www.biomedcentral.com/1472-6963/13/76/prepub

## Supplementary Material

Additional file 1 **Specially designed algorithms for risk and effectiveness assessment used in the IMPACT trial.** The algorithms are based on the same stratifications as the SCORE model and present the estimated ten-year mortality risk and the remaining life expectancy, respectively, without statin treatment as well as the estimated ARR in percentages and POL in months, respectively, if treated with a statin. Participating GPs were allocated and instructed to use only one of the respective effectiveness formats when informing their patients about CVD risk and the effectiveness of statin therapy. Click here for file
