# Supplementary material for: Impact of effectiveness information format on patient choice of therapy and satisfaction with decisions about chronic disease medication: the "Influence of intervention Methodologies on Patient Choice of Therapy (IMPACT)" cluster-randomised trial in general practice
Source: BMC Health Serv Res. 2013 Feb 25;13:76. doi: 10.1186/1472-6963-13-76 (PMC3599428; doi:10.1186/1472-6963-13-76)
Supplement: Additional file 1 — Specially designed algorithms for risk and effectiveness assessment used in the IMPACT trial. The algorithms are based on the same stratifications as the SCORE model and present the estimated ten-year mortality risk and the remaining life expectancy, respectively, without statin treatment as well as the estimated ARR in percentages and POL in months, respectively, if treated with a statin. Participating GPs were allocated and instructed to use only one of the respective effectiveness formats when informing their patients about CVD risk and the effectiveness of statin therapy. [file 1472-6963-13-76-S1.pdf]

WOMEN

10 YEARS' MORTALITY RISK (%)

***without** treatment with cholesterol lowering drug*

40 YEARS

SYST.BP	NON-SMOKER					SMOKER				
	4	5	6	7	8	4	5	6	7	8
180	0.2	0.2	0.2	0.3	0.3	0.3	0.4	0.4	0.5	0.6
160	0.1	0.1	0.1	0.2	0.2	0.2	0.3	0.3	0.3	0.4
140	0.1	0.1	0.1	0.1	0.1	0.1	0.2	0.2	0.2	0.3
120	0.1	0.1	0.1	0.1	0.1	0.1	0.1	0.1	0.2	0.2

TOTAL CHOLESTEROL (mmol/l)

50 YEARS

SYST.BP	NON-SMOKER					SMOKER				
	4	5	6	7	8	4	5	6	7	8
180	0.9	1.1	1.2	1.4	1.7	1.8	2.1	2.4	2.8	3.3
160	0.6	0.7	0.8	1.0	1.2	1.2	1.4	1.6	1.9	2.3
140	0.4	0.5	0.6	0.7	0.8	0.8	1.0	1.1	1.3	1.6
120	0.3	0.3	0.4	0.5	0.5	0.6	0.6	0.8	0.9	1.1

TOTAL CHOLESTEROL (mmol/l)

55 YEARS

SYST.BP	NON-SMOKER					SMOKER				
	4	5	6	7	8	4	5	6	7	8
180	1.9	2.1	2.4	2.8	3.3	3.6	4.1	4.7	5.5	6.5
160	1.3	1.4	1.7	1.9	2.3	2.4	2.8	3.2	3.8	4.5
140	0.8	1.0	1.1	1.3	1.6	1.6	1.9	2.2	2.6	3.1
120	0.6	0.7	0.8	0.9	1.1	1.1	1.3	1.5	1.8	2.1

TOTAL CHOLESTEROL (mmol/l)

60 YEARS

SYST.BP	NON-SMOKER					SMOKER				
	4	5	6	7	8	4	5	6	7	8
180	3.4	3.9	4.5	5.2	6.1	6.6	7.5	8.6	10	12
160	2.3	2.6	3.0	3.5	4.2	4.5	5.1	5.9	6.9	8.1
140	1.6	1.8	2.1	2.4	2.9	3.0	3.5	4.0	4.7	5.6
120	1.0	1.2	1.4	1.6	2.0	2.0	2.4	2.7	3.2	3.9

TOTAL CHOLESTEROL (mmol/l)

65 YEARS

SYST.BP	NON-SMOKER					SMOKER				
	4	5	6	7	8	4	5	6	7	8
180	5.9	6.7	7.6	8.8	10	11	13	14	17	19
160	4.0	4.5	5.2	6.1	7.1	7.6	8.7	10	12	14
140	2.7	3.1	3.5	4.1	4.9	5.2	5.9	6.9	8.0	9.5
120	1.8	2.1	2.4	2.8	3.4	3.5	4.0	4.7	5.5	6.6

TOTAL CHOLESTEROL (mmol/l)

10 YEARS' ABSOLUTE RISK REDUCTION (ARR) IN MORTALITY RISK (%)

***With lifelong treatment** with cholesterol lowering drug*

40 YEARS

SYST.BP	NON-SMOKER					SMOKER				
	4	5	6	7	8	4	5	6	7	8
180	0.0	0.0	0.0	0.0	0.0	0.0	0.1	0.1	0.1	0.1
160	0.0	0.0	0.0	0.0	0.0	0.0	0.0	0.0	0.0	0.1
140	0.0	0.0	0.0	0.0	0.0	0.0	0.0	0.0	0.0	0.0
120	0.0	0.0	0.0	0.0	0.0	0.0	0.0	0.0	0.0	0.0

TOTAL CHOLESTEROL (mmol/l)

50 YEARS

SYST.BP	NON-SMOKER					SMOKER				
	4	5	6	7	8	4	5	6	7	8
180	0.1	0.1	0.2	0.2	0.2	0.3	0.3	0.3	0.4	0.5
160	0.1	0.1	0.1	0.1	0.2	0.2	0.2	0.2	0.3	0.3
140	0.1	0.1	0.1	0.1	0.1	0.1	0.1	0.2	0.2	0.2
120	0.0	0.0	0.1	0.1	0.1	0.1	0.1	0.1	0.1	0.1

TOTAL CHOLESTEROL (mmol/l)

55 YEARS

SYST.BP	NON-SMOKER					SMOKER				
	4	5	6	7	8	4	5	6	7	8
180	0.3	0.3	0.3	0.4	0.4	0.5	0.5	0.6	0.7	0.9
160	0.2	0.2	0.2	0.3	0.3	0.3	0.4	0.4	0.5	0.6
140	0.1	0.1	0.2	0.2	0.2	0.2	0.3	0.3	0.3	0.4
120	0.1	0.1	0.1	0.1	0.1	0.1	0.2	0.2	0.2	0.3

TOTAL CHOLESTEROL (mmol/l)

60 YEARS

SYST.BP	NON-SMOKER					SMOKER				
	4	5	6	7	8	4	5	6	7	8
180	0.4	0.5	0.6	0.7	0.8	0.8	0.9	1.1	1.2	1.4
160	0.3	0.3	0.4	0.5	0.5	0.6	0.7	0.7	0.9	1.0
140	0.2	0.2	0.3	0.3	0.4	0.4	0.4	0.5	0.6	0.7
120	0.1	0.2	0.2	0.2	0.3	0.3	0.3	0.4	0.4	0.5

TOTAL CHOLESTEROL (mmol/l)

65 YEARS

SYST.BP	NON-SMOKER					SMOKER				
	4	5	6	7	8	4	5	6	7	8
180	0.7	0.8	0.9	1.0	1.2	1.3	1.5	1.6	1.9	2.1
160	0.5	0.5	0.6	0.7	0.8	0.9	1.0	1.2	1.4	1.6
140	0.3	0.4	0.4	0.5	0.6	0.6	0.7	0.8	1.0	1.1
120	0.2	0.3	0.3	0.3	0.4	0.4	0.5	0.6	0.7	0.8

TOTAL CHOLESTEROL (mmol/l)

MEN

10 YEARS' MORTALITY RISK (%)
***without** treatment with cholesterol lowering drug*

10 YEARS' ABSOLUTE RISK REDUCTION (ARR) IN MORTALITY RISK (%)
***With lifelong treatment** with cholesterol lowering drug*

40 YEARS

Mortality risk (%) without treatment:

SYST.BP	NON-SMOKER 4	5	6	7	8	SMOKER 4	5	6	7	8
180	0.7	0.9	1.1	1.3	1.6	1.4	1.8	2.1	2.6	3.2
160	0.5	0.6	0.7	0.9	1.1	1.0	1.2	1.5	1.8	2.2
140	0.3	0.4	0.5	0.6	0.8	0.7	0.8	1.0	1.3	1.6
120	0.2	0.3	0.4	0.4	0.5	0.5	0.6	0.7	0.9	1.1

TOTAL-CHOLESTEROL (mmol/l)

→

ARR (%) with lifelong treatment:

SYST.BP	NON-SMOKER 4	5	6	7	8	SMOKER 4	5	6	7	8
180	0.1	0.2	0.2	0.2	0.3	0.3	0.3	0.4	0.5	0.6
160	0.1	0.1	0.1	0.2	0.2	0.2	0.2	0.3	0.3	0.4
140	0.1	0.1	0.1	0.1	0.1	0.1	0.1	0.2	0.2	0.3
120	0.0	0.1	0.1	0.1	0.1	0.1	0.1	0.1	0.2	0.2

TOTAL-CHOLESTEROL (mmol/l)

50 YEARS

SYST.BP	NON-SMOKER 4	5	6	7	8	SMOKER 4	5	6	7	8
180	2.6	3.0	3.7	4.4	5.4	5.0	6.0	7.2	8.7	11
160	1.7	2.1	2.5	3.1	3.8	3.4	4.2	5.0	6.1	7.4
140	1.2	1.4	1.7	2.1	2.6	2.4	2.8	3.5	4.2	5.2
120	0.8	1.0	1.2	1.5	1.8	1.6	2.0	2.4	2.9	3.6

TOTAL-CHOLESTEROL (mmol/l)

→

SYST.BP	NON-SMOKER 4	5	6	7	8	SMOKER 4	5	6	7	8
180	0.4	0.5	0.6	0.7	0.9	0.8	1.0	1.2	1.4	1.7
160	0.3	0.4	0.4	0.5	0.6	0.6	1.7	0.8	1.0	1.2
140	0.2	0.2	0.3	0.4	0.4	0.4	0.5	0.6	0.7	0.9
120	0.1	0.2	0.2	0.3	0.3	0.3	0.3	0.4	0.5	0.6

TOTAL-CHOLESTEROL (mmol/l)

55 YEARS

SYST.BP	NON-SMOKER 4	5	6	7	8	SMOKER 4	5	6	7	8
180	4.2	5.0	6.0	7.2	8.7	8.2	9.7	12	14	17
160	2.9	3.4	4.1	5.0	6.1	5.7	6.7	8.1	9.7	12
140	2.0	2.4	2.8	3.5	4.2	3.9	4.7	5.6	6.8	8.3
120	1.4	1.6	2.0	2.4	2.9	2.7	3.2	3.9	4.7	5.8

TOTAL-CHOLESTEROL (mmol/l)

→

SYST.BP	NON-SMOKER 4	5	6	7	8	SMOKER 4	5	6	7	8
180	0.7	0.8	1.0	1.1	1.4	1.3	1.5	1.8	2.1	2.4
160	0.5	0.6	0.7	0.8	1.0	0.9	1.1	1.3	1.5	1.8
140	0.3	0.4	0.5	0.6	0.7	0.6	0.8	0.9	1.1	1.3
120	0.2	0.3	0.3	0.4	0.5	0.4	0.5	0.6	0.8	0.9

TOTAL-CHOLESTEROL (mmol/l)

60 YEARS

SYST.BP	NON-SMOKER 4	5	6	7	8	SMOKER 4	5	6	7	8
180	6.6	7.7	9.2	11	13	13	15	17	21	25
160	4.5	5.3	6.4	7.6	9.2	8.7	10	12	15	18
140	3.1	3.7	4.4	5.3	6.5	6.0	7.2	8.6	10	13
120	2.1	2.5	3.0	3.7	4.5	4.2	5.0	6.0	7.2	8.8

TOTAL-CHOLESTEROL (mmol/l)

→

SYST.BP	NON-SMOKER 4	5	6	7	8	SMOKER 4	5	6	7	8
180	1.0	1.2	1.4	1.6	1.9	1.8	2.1	2.4	2.7	3.1
160	0.7	0.8	1.0	1.2	1.4	1.3	1.5	1.8	2.1	2.4
140	0.5	0.6	0.7	0.8	1.0	0.9	1.1	1.3	1.5	1.8
120	0.3	0.4	0.5	0.6	0.7	0.6	0.8	0.9	1.1	1.3

TOTAL-CHOLESTEROL (mmol/l)

65 YEARS

SYST.BP	NON-SMOKER 4	5	6	7	8	SMOKER 4	5	6	7	8
180	9.7	11	13	16	19	18	21	25	29	34
160	6.7	7.9	9.3	11	13	13	15	18	21	25
140	4.6	5.4	6.5	7.8	9.4	8.9	11	13	15	18
120	3.2	3.7	4.5	5.4	6.6	6.2	7.3	8.8	11	13

TOTAL-CHOLESTEROL (mmol/l)

→

SYST.BP	NON-SMOKER 4	5	6	7	8	SMOKER 4	5	6	7	8
180	1.3	1.5	1.8	2.0	2.4	2.3	2.6	2.9	3.2	3.6
160	0.9	1.1	1.3	1.5	1.8	1.7	2.0	2.2	2.6	2.9
140	0.7	0.8	0.9	1.1	1.3	1.2	1.4	1.7	1.9	2.3
120	0.5	0.5	0.6	0.8	0.9	0.9	1.0	1.2	1.4	1.7

TOTAL-CHOLESTEROL (mmol/l)

WOMEN

LIFE EXPECTANCY (YEARS)
<u>without</u> treatment with cholesterol lowering drug

40 YEARS

SYST.BP	NON-SMOKER					SMOKER				
180	41	40	40	39	39	38	38	37	36	35
160	42	41	41	40	40	40	39	39	38	37
140	42	42	42	41	41	41	41	40	40	39
120	43	43	42	42	42	42	42	41	41	40
	4	5	6	7	8	4	5	6	7	8

TOTAL CHOLESTEROL (mmol/l)

50 YEARS

SYST.BP	NON-SMOKER					SMOKER				
180	31	31	30	30	29	29	28	28	27	26
160	32	32	31	31	31	30	30	29	29	28
140	33	33	32	32	32	32	31	31	30	29
120	33	33	33	33	33	32	32	32	31	31
	4	5	6	7	8	4	5	6	7	8

TOTAL CHOLESTEROL (mmol/l)

55 YEARS

SYST.BP	NON-SMOKER					SMOKER				
180	27	26	26	25	25	24	24	23	22	22
160	28	27	27	27	26	26	25	25	24	23
140	28	28	28	28	27	27	27	26	26	25
120	29	29	29	28	28	28	28	27	27	26
	4	5	6	7	8	4	5	6	7	8

TOTAL CHOLESTEROL (mmol/l)

60 YEARS

SYST.BP	NON-SMOKER					SMOKER				
180	22	22	22	21	21	20	20	19	18	18
160	23	23	23	22	22	22	21	21	20	19
140	24	24	24	23	23	23	22	22	21	21
120	25	24	24	24	24	24	23	23	23	22
	4	5	6	7	8	4	5	6	7	8

TOTAL CHOLESTEROL (mmol/l)

65 YEARS

SYST.BP	NON-SMOKER					SMOKER				
180	18	18	18	17	17	16	16	15	15	14
160	19	19	19	18	18	18	17	17	16	16
140	20	20	20	19	19	19	18	18	18	17
120	21	20	20	20	20	20	19	19	19	18
	4	5	6	7	8	4	5	6	7	8

TOTAL CHOLESTEROL (mmol/l)

PROLONGATION OF LIFE (POL)(MONTHS)
<u>With lifelong treatment</u> with cholesterol lowering drug

40 YEARS

SYST.BP	NON-SMOKER					SMOKER				
180	5	5	5	6	7	7	7	8	8	9
160	3	4	4	5	5	5	6	6	7	8
140	2	3	3	4	4	4	5	5	6	6
120	2	2	2	3	3	3	3	4	4	5
	4	5	6	7	8	4	5	6	7	8

TOTAL CHOLESTEROL (mmol/l)

50 YEARS

SYST.BP	NON-SMOKER					SMOKER				
180	5	5	5	6	6	7	7	8	8	9
160	3	4	4	5	5	5	6	6	7	7
140	2	3	3	4	4	4	5	5	6	6
120	2	2	2	3	3	3	3	4	4	5
	4	5	6	7	8	4	5	6	7	8

TOTAL CHOLESTEROL (mmol/l)

55 YEARS

SYST.BP	NON-SMOKER					SMOKER				
180	4	5	5	6	6	7	7	7	8	8
160	3	4	4	5	5	5	6	6	7	7
140	2	3	3	3	4	4	5	5	6	6
120	2	2	2	3	3	3	3	4	4	5
	4	5	6	7	8	4	5	6	7	8

TOTAL CHOLESTEROL (mmol/l)

60 YEARS

SYST.BP	NON-SMOKER					SMOKER				
180	4	5	5	6	6	6	7	7	8	8
160	3	4	4	4	5	5	6	6	6	7
140	2	3	3	3	4	4	4	5	5	6
120	2	2	2	2	3	3	3	4	4	5
	4	5	6	7	8	4	5	6	7	8

TOTAL CHOLESTEROL (mmol/l)

65 YEARS

SYST.BP	NON-SMOKER					SMOKER				
180	4	4	5	5	6	6	6	7	7	7
160	3	3	4	4	5	5	5	6	6	6
140	2	3	3	3	4	4	4	5	5	5
120	2	2	2	2	3	3	3	4	4	4
	4	5	6	7	8	4	5	6	7	8

TOTAL CHOLESTEROL (mmol/l)

MEN

LIFE EXPECTANCY (YEARS)

***without* treatment with cholesterol lowering drug**

40 YEARS

SYST.BP	NON-SMOKER					SMOKER				
180	35	34	34	33	32	32	31	30	29	27
160	36	36	35	34	34	34	33	32	31	30
140	38	37	37	36	35	35	35	34	33	32
120	38	38	38	37	37	37	36	36	35	34
	4	5	6	7	8	4	5	6	7	8

TOTAL CHOLESTEROL (mmol/l)

50 YEARS

SYST.BP	NON-SMOKER					SMOKER				
180	26	26	25	24	23	23	22	21	20	19
160	28	27	26	26	25	25	24	23	22	21
140	28	28	28	27	26	27	26	25	24	23
120	29	29	29	28	28	28	27	27	26	25
	4	5	6	7	8	4	5	6	7	8

TOTAL CHOLESTEROL (mmol/l)

55 YEARS

SYST.BP	NON-SMOKER					SMOKER				
180	22	21	21	20	19	19	18	17	16	15
160	23	23	22	22	21	21	20	19	18	17
140	24	24	23	23	22	22	22	21	20	19
120	25	25	24	24	23	24	23	23	22	21
	4	5	6	7	8	4	5	6	7	8

TOTAL CHOLESTEROL (mmol/l)

60 YEARS

SYST.BP	NON-SMOKER					SMOKER				
180	18	18	17	16	16	16	15	14	13	12
160	19	19	18	18	17	17	17	16	15	14
140	20	20	20	19	18	19	18	17	17	16
120	21	21	20	20	20	20	19	19	18	17
	4	5	6	7	8	4	5	6	7	8

TOTAL CHOLESTEROL (mmol/l)

65 YEARS

SYST.BP	NON-SMOKER					SMOKER				
180	15	14	14	13	12	13	12	11	10	9
160	16	16	15	15	14	14	13	13	12	11
140	17	16	16	16	15	15	15	14	13	13
120	17	17	17	16	16	16	16	15	15	14
	4	5	6	7	8	4	5	6	7	8

TOTAL CHOLESTEROL (mmol/l)

PROLONGATION OF LIFE (POL)(MONTHS)

***With lifelong treatment* with cholesterol lowering drug**

40 YEARS

SYST.BP	NON-SMOKER					SMOKER				
180	6	7	8	9	9	9	10	10	11	11
160	5	5	6	7	8	8	8	9	10	10
140	4	4	5	5	6	6	7	7	8	9
120	3	3	4	4	5	5	5	6	7	8
	4	5	6	7	8	4	5	6	7	8

TOTAL CHOLESTEROL (mmol/l)

50 YEARS

SYST.BP	NON-SMOKER					SMOKER				
180	6	7	7	8	9	9	9	10	10	10
160	5	5	6	7	7	7	8	9	10	10
140	3	4	5	5	6	6	6	7	8	9
120	3	3	3	4	5	4	5	6	6	7
	4	5	6	7	8	4	5	6	7	8

TOTAL CHOLESTEROL (mmol/l)

55 YEARS

SYST.BP	NON-SMOKER					SMOKER				
180	6	6	7	8	8	8	9	9	9	10
160	4	5	6	6	7	7	7	8	9	9
140	3	4	4	5	6	5	6	7	7	8
120	2	3	3	4	4	4	5	5	6	7
	4	5	6	7	8	4	5	6	7	8

TOTAL CHOLESTEROL (mmol/l)

60 YEARS

SYST.BP	NON-SMOKER					SMOKER				
180	5	6	6	7	8	7	8	8	8	9
160	4	5	5	6	6	6	7	7	8	8
140	3	4	4	5	5	5	6	6	7	7
120	2	3	3	3	4	4	4	5	6	6
	4	5	6	7	8	4	5	6	7	8

TOTAL CHOLESTEROL (mmol/l)

65 YEARS

SYST.BP	NON-SMOKER					SMOKER				
180	5	5	6	6	7	7	7	7	7	7
160	4	4	5	5	6	6	6	6	7	7
140	3	3	4	4	5	5	5	5	6	7
120	2	2	3	3	4	3	4	4	5	6
	4	5	6	7	8	4	5	6	7	8

TOTAL CHOLESTEROL (mmol/l)
